# Phylogenetic analysis of the complete mitochondrial genome of the Japanese peacock butterfly *Aglais io geisha* (Stichel 1907) (Insecta: Lepidoptera: Nymphalidae)

**DOI:** 10.1080/23802359.2021.1981168

**Published:** 2021-09-27

**Authors:** Mackenzie R. Alexiuk, Melanie M. L. Lalonde, Jeffrey M. Marcus

**Affiliations:** Department of Biological Sciences, University of Manitoba, Winnipeg, Canada

**Keywords:** Illumina sequencing, mitogenomics, Lepidoptera, Nymphalidae, Nymphalini

## Abstract

The peacock butterfly *Aglais io* (Linnaeus, 1758) (Nymphalidae: Nymphalinae: Nymphalini) is a colorful and charismatic flagship butterfly species whose range spans from the British Isles and Europe through temperate Asia and the Far East. In Europe, it has been used as a model species for studying the effects of GMO maize pollen on caterpillar growth and survivorship. The Japanese subspecies, *Aglais io geisha* (Stichel 1907), is not as well studied as its European counterpart. Genome skimming by Illumina sequencing allowed the assembly of a complete circular mitochondrial genome (mitogenome) of 15,252 bp from *A. io geisha* consisting of 80.6% AT nucleotides, 13 protein-coding genes, 22 tRNAs, two rRNAs, and a control region in the gene order typical of butterfly species. *Aglais io geisha COX1* gene features an atypical start codon (CGA) while *COX1, COX2, CYTB, ND1, ND3*, *ND4,* and *ND5* display incomplete stop codons finished by the addition of 3’ A residues to the mRNA. Bayesian phylogenetic reconstruction places *A. io geisha* within a clade with European *A. io* mitogenomes in the tribe Nymphalini, which is consistent with previous phylogenetic hypotheses.

The peacock butterfly *Aglais io* (Linnaeus, 1758) (Nymphalidae: Nymphalinae: Nymphalini) is an indicator species for studying the effects of GMO maize pollen on non-targeted insects in Europe (Arpaia et al. 2018; Leclerc et al. 2018). The natural range of *A. io* includes the British Isles, Europe, temperate Asia, and the Far East, but has recently expanded its range into North America (Nazari et al. 2018).

*Aglais io* is a colorful bivoltine species producing two broods of offspring, with one that flies in summer and one that over-winters as adults (Arpaia et al. 2018; Leclerc et al. 2018). The adults feed on nectar-bearing plants, while the caterpillars feed on members of the nettle family Urticaceae which is the route by which GMO maize pollen is ingested (Leclerc et al. 2018). Adult females lay eggs in large pyramidal clusters on hops plants (*Humulus lupulus)* to protect the innermost layer of eggs from parasitism by flies (Tachnidae) and wasps (Ichneumonidae) (Hondo et al. 1995; Audusseau et al. 2021). Adults have been observed to fake death upon wings being pinched together, with antennae becoming immobile and legs stiffening against the body (Loxdale 2017). Adults also have sound-producing eye-spots on their wings to deter predators including bats and birds (Møhl and Miller 1976; Vallin et al. 2005; Loxdale 2017). Less is known about the focus of the current study, the Japanese peacock butterfly, subspecies *A. io geisha* (Stichel 1907), than its European counterpart *A. io*.

Here we report the complete mitochondrial genome (mitogenome) sequence of *A. io geisha* from specimen Ai2015.2, collected in Saitama, Japan (GPS 35.90807 N, 139.65657E) in July 2015 that has been pinned, spread, and deposited in the Wallis Roughley Museum of Entomology, University of Manitoba (http://www.wallisroughley.ca/, Jason Gibbs, Jason.Gibbs@umanitoba.ca) voucher WRME0507739.

DNA was prepared (McCullagh and Marcus 2015) and later sequenced by Illumina NovaSeq6000 (San Diego, CA) (Marcus 2018). The mitogenome of *A. io geisha* (Genbank MZ322948) was assembled and annotated using Geneious Prime 2021.1 from an SRA library of 23,191,042 paired 150 bp reads (Genbank SRA PRJNA733565) using *Aglais io* and *Araschnia levana* reference mitogenomes (Lepidoptera: Nymphalidae, KM592970; Lepidoptera: Nymphalidae, MT712075) (Timmermans et al. 2016; Alexiuk et al. 2020a). The *A. io geisha* nuclear rRNA repeat (Genbank MZ322949) was also assembled and annotated using an *A. levana* (MT750296) reference sequence. The rRNA repeat sequence is increasingly recognized as being very useful for phylogenetic comparisons based on nuclear markers (Dodsworth 2015; Coissac et al. 2016; Marcus 2018; Krehenwinkel et al. 2019), so we have chosen to release it here.

The *A. io geisha* circular 15,252 bp mitogenome assembly was composed of 2700 paired reads with nucleotide composition: 40.1% A, 11.9% C, 7.5% G, and 40.5% T. The gene composition and order in *A. io geisha* is typical of the arrangement found in most butterfly mitogenomes (Park et al. 2016). The *A. io geisha* protein-coding gene start codons include ATG (*ATP6, COX2, COX3, CYTB, ND1, ND4*), ATT (*ND2, ND3, ND5*), ATC, (*ND6*), CGA, an atypical *COX1* start codon that is also found in the *COX1* gene of many other insects (Liao et al. 2010). Additionally, *ATP8* and *ND4L* have ATA start codons that are infrequently used in insect mitochondria but are frequently used in other animal groups (Okimoto et al. 1990; Han et al. 2016; Alexiuk et al. 2020b). The mitogenome contains five protein-coding genes *(COX1, COX2, CYTB, ND3, ND5)* with single-nucleotide (T––) stop codons, and two protein-coding genes (*ND1, ND4*) with two-nucleotide (TA–) stop codons completed by post-transcriptional addition of 3′ A residues. All structures of the tRNAs were verified using ARWEN v.1.2 (Laslett and Canback 2008) and have typical cloverleaf secondary structures with the exception for trnS (AGN) where the dihydrouridine arm is replaced by a loop, whereas the control region and mitochondrial rRNAs are typical for Lepidoptera (McCullagh and Marcus 2015).

Phylogenetic reconstruction (Figure 1) was completed using the complete mitogenome of *A. io geisha,* 34 mitogenomes from the subfamily Nymphalinae and three outgroup species from subfamily Limenitidinae (Alexiuk et al. 2020b; Hamilton et al. 2020; Lalonde and Marcus 2020; Payment et al. 2020; Lalonde 2021; Alexiuk et al. 2021). Mitogenome sequences were aligned in CLUSTALX 2.1 (Thompson et al. 1997; Larkin et al. 2007) and analyzed using Bayesian Inference with the GTR + I + G model (model selected using jModeltest 2.1.1 (Darriba et al. 2012)) in Mr. Bayes version 3.2.7 (Ronquist and Huelsenbeck 2003; Ronquist et al. 2012). As expected based on previous phylogenetic hypotheses (Nylin et al. 2001; Wahlberg and Nylin 2003; Wahlberg et al. 2009), phylogenetic analysis places *A. io geisha* in a clade with mitogenomes from European *A. io* specimens within tribe Nymphalini.

**Figure 1.**
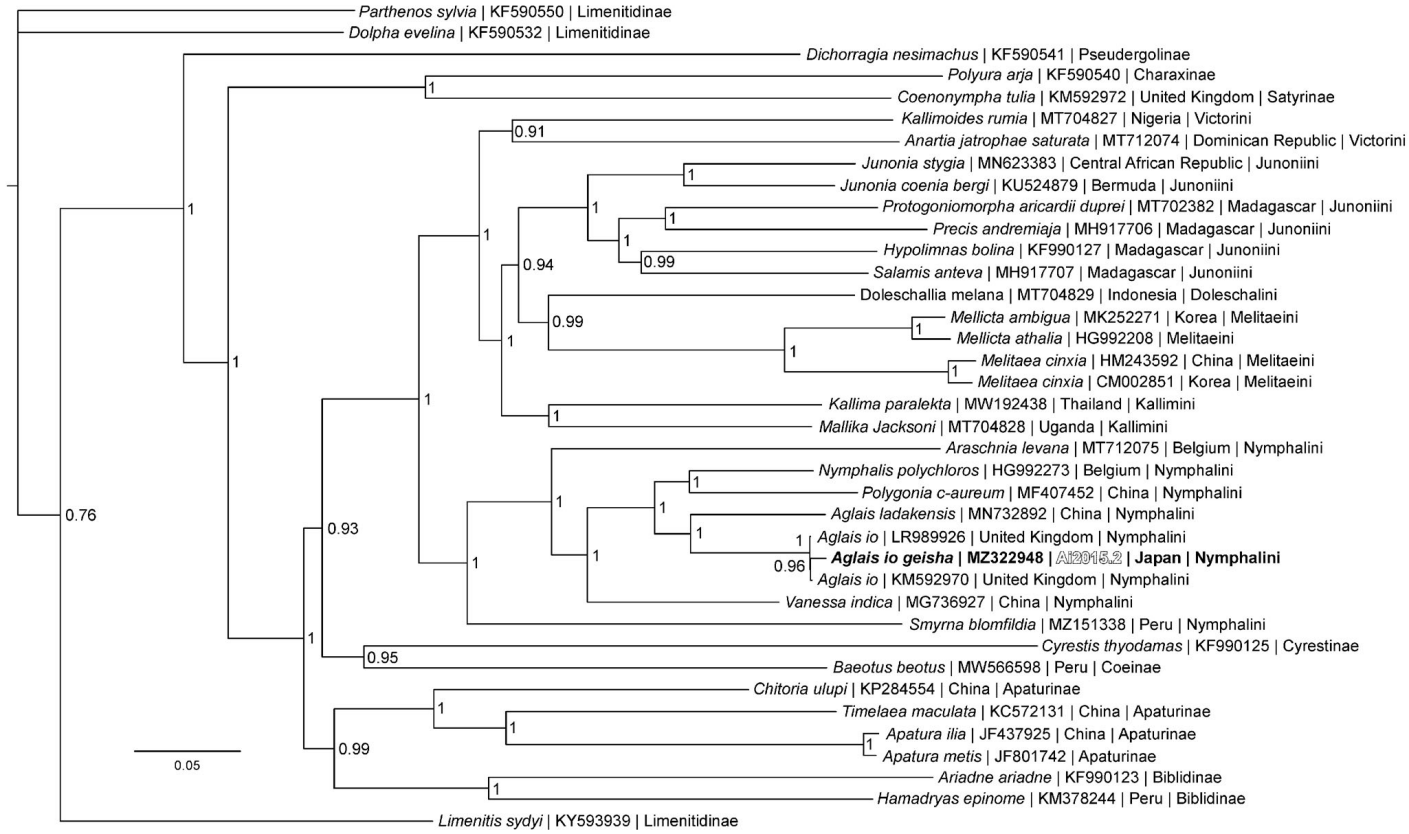
The Bayesian phylogeny (GTR + I + G model, average Potential Scale Reduction Factor (PSRF) = 1, average deviation of split frequencies = 0.000628) of the *Aglais io geisha* mitogenome, 37 additional mitogenomes from within family Nymphalidae, including outgroup species *Limenitis sydyi*, *Parthenos sylvia*, and *Dophla evelina* (Limenitinae) (Alexiuk et al. 2020b; Hamilton et al. 2020; Lalonde and Marcus 2020; Payment et al. 2020; Lalonde 2021), produced by 10 million MCMC generations in MrBayes, with sampling every 100 generations, and after discarding the first 250,000 generations as burn-in. The Bayesian posterior probability values determined by MrBayes are provided at each node.

## Data Availability

The genome sequence data that support the findings of this study are openly available in GenBank of NCBI at [https://www.ncbi.nlm.nih.gov] (https://www.ncbi.nlm.nih.gov/) under the accession nos. MZ322948 and MZ322949. The associated BioProject, SRA, and Bio-Sample numbers are PRJNA733565, SRX11064013, and SAMN19415664 respectively.
